# Effectiveness of a low-value financial-incentive program for increasing vegetable-rich restaurant meal selection and reducing socioeconomic inequality: a cluster crossover trial

**DOI:** 10.1186/s12966-019-0830-5

**Published:** 2019-09-12

**Authors:** Wataru Nagatomo, Junko Saito, Naoki Kondo

**Affiliations:** 1 Department of Health Education and Health Sociology, School of Public Health, The University of Tokyo, 7-3-1 Hongo, Bunkyo-ku, Tokyo, 113-0033 Japan; 2 Division of Prevention, Centre for Public Health Sciences, National Cancer Centre, 5-1-1 Tsukiji, Chuo-ku, Tokyo, 104-0045 Japan

**Keywords:** Health inequality, Diet, Health behaviour, Marketing, Nudge, Japan

## Abstract

**Background:**

In light of recent theories in behavioural economics, an intervention program with monetary incentives could be effective for helping patrons order healthy food, even if the incentive is small and less than one’s perceived marginal value.

**Methods:**

In this single-arm cluster crossover trial at 26 local restaurants, a 1-week campaign offered a 50-yen (approximately 0.5 US dollars) cash-back payment to customers ordering vegetable-rich meals, while no pre-order incentives were offered during the control period.

**Results:**

In total, 511 respondents out of 7537 customers (6.8%), and 704 respondents out of 7826 customers (9.0%), ordered vegetable-rich meals during the control and intervention periods, respectively. During the intervention period, the covariate-adjusted proportion of vegetable-rich meal orders was 1.50 times higher (95% confidence interval [CI]: 1.29 to 1.75), which increased daily sales by 1.77 times (95% CI: 1.11 to 2.83), even when subtracting the cost of cash-back payments. Respondents who reported spending the least amount of money on eating out (used as a proxy measure for income) were the least likely to order vegetable-rich meals during the control period. However, these individuals increased their proportion of purchasing such meals during the intervention period (a 3.8 percentage point increase (95% CI: 2.82 to 4.76) among those spending the least vs a 2.1 percentage point increase (95% CI: 1.66 to 2.62) among those spending the most; *P* for interaction = 0.001). Similarly, irregular employees exhibited a larger increase (+ 5.2 percentage points, 95% CI: 4.54 to 5.76) than did regular workers (− 1.4, 95% CI: − 1.66 to − 1.05, *P* for interaction = 0.001).

**Conclusions:**

A program with an immediate low-value monetary incentive could be a public health measure for reducing inequalities in making healthy food choices.

**Trial registration:**

UMIN Clinical Trials Registry, UMIN000022396. Registered 21 May 2016.

**Electronic supplementary material:**

The online version of this article (10.1186/s12966-019-0830-5) contains supplementary material, which is available to authorized users.

## Background

Non-communicable diseases (NCDs) constitute a global health issue, accounting for two-thirds of deaths worldwide [1, 2]. In many countries, NCD-related deaths and their behavioural risks, such as smoking, drinking, and insufficient intake of vegetables and fruit, are more frequent among people with low socioeconomic status (SES) compared to those with high SES [3–9]. Given that individual SES is largely attributable to structural factors in society, national policies for providing financial protections, including income redistribution measures and universal healthcare coverage, are essential [10–12]. Nonetheless, health gaps clearly exist, even in countries offering the most affordable welfare services [1]. Alternative approaches to promote healthy behaviours are needed. Such interventions should be evaluated for their effectiveness among socially disadvantaged groups [2, 3], but the evidence is still limited.

In recent years, much attention has been paid to the notion of encouraging people to make favourable choices while safeguarding the individual’s discretion to make his or her own decisions [13–15]. Price changes of unhealthy and healthy goods and services (e.g. tobacco and sugar-sweetened beverages) is a popular and effective measure [16–18]. As people with lower incomes tend to focus on prices when choosing foods, price changes could be a useful public health measure for reducing socioeconomic inequality in regards to behaviour choices [19–21]. However, existing research has mainly evaluated the efficacy of such measures in a laboratory environment [22, 23], and few studies have evaluated the effectiveness of positive monetary incentives for reducing socioeconomic inequality regarding healthy food choices in real-world settings [24]. Importantly, price change interventions could be effective, even though the difference between new and old prices may not be sufficient to fully overcome the difference in utility to consumers. For example, although the size of a discount may not be in line with consumers’ actual willingness to pay for the good(s) in question, individuals may still purchase the item(s), due to biases in human cognition and behaviour, including framing effects and loss aversion [25]. Therefore, we conducted an intervention study, within a real-world setting, whereby a “small cash back” measure was implemented to encourage people to select vegetable-rich meals from menus at participating restaurants. We tested three hypotheses: (1) the intervention will increase overall vegetable-rich meal orders; (2) restaurants’ total sales will increase; and most importantly, (3) vegetable-rich menu orders will increase in proportion to the individual’s level of social disadvantage.

## Methods

### Research setting and design

The government of Adachi Ward, a business and residential area in the northern Tokyo metropolitan area, runs a program entitled “Eat Vegetables Daily” (in Japanese: *Adachi beji-tabe-raifu*). The program was put in place as part of a set of type 2 diabetes countermeasures [26–29] strengthening environmental interventions for the communities, rather than providing health educational opportunities for individuals [26, 27]. With this program, the government has sought collaborations with nearly 600 local restaurants and retail shops. Within these locales, “vegetable-rich” meals are served. For a shop or restaurant to be certified as a partner, it must develop and serve vegetable-rich meals containing at least 120 g or more of vegetables per serving (except potatoes). In June 2016, we conducted a single-arm cluster crossover trial at local restaurants. The inclusion criteria were that the restaurant was already participating in the program and that the restaurant accepted an invitation from the Adachi government to participate in this study. We obtained informed consent, asking customers not to answer the questionnaire if they were not willing to participate in the study. We excluded those who were unable to communicate in Japanese or read the survey’s participation agreement form.

Two continuous weeks were designated as the data collection period: the first week was the control period, and the second week was the intervention period. We allocated each cluster, a restaurant, to both the control and intervention periods. During the intervention period, we implemented a cash-back campaign in which 50 yen were refunded upon payment to any customer ordering a vegetable-rich meal (a US dollar equalled 105.49 yen in June 2016 [30]). During the intervention period, the participating restaurants displayed posters informing customers that the campaign was underway and placed vegetable-rich menus in front of their entrances so that customers could sufficiently account for the incentives provided by the campaign before ordering. To prevent the restaurant owners from displaying the poster before the intervention period, we sent an advertising poster to be displayed in the front of the restaurant a few days before the intervention period. We also provided oral and written guidance, requesting that restaurant owners display the poster on the morning of the first day of the campaign and to avoid displaying it during the pre-campaign period.

To maximise data accuracy, feasibility, and practicality in a real-world setting, we did not conduct a parallel-group randomised trial. Compared to a simple cluster randomised trial, a single-arm crossover design has the advantage of completely matching the restaurants’ characteristics between the control and intervention periods. The Research Ethics Committee of the Faculty of Medicine at the University of Tokyo (UMIN Clinical Trials Registry number: UMIN000022396) approved this protocol.

### Data collection

During the control and intervention periods, we conducted surveys using self-administered questionnaires, with two different sets of targets and protocols. During the intervention period, a 50-yen cash-back incentive was offered for ordering a vegetable-rich meal, but this payment was conditional on submitting a completed questionnaire sheet. To collect information on individuals who ordered vegetable-rich meals, the first survey targeted all customers who ordered such meals during both the control and the intervention periods (survey A). For survey A, we trained restaurant staff members on how to conduct all survey procedures, including distributing and gathering questionnaires and paying out the cash-back incentive. To avoid imbalances in questionnaire response rates between the intervention and control periods, we also offered cash back to those who ordered vegetable-rich meals during the control period. However, during the control period, information regarding the cash-back incentive was not provided to customers until they had decided on their order; thus, this information was provided *after* the order was placed.

To acquire data on the total number of customers based on SES, we conducted another survey targeting all customers, including those who did not order vegetable-rich meals (survey B). We surveyed customers who visited during a randomly selected one-hour period during both the control and intervention periods at each restaurant.

We also asked the restaurant owners to provide information regarding their daily sales. Because meal selection was expected to be influenced by the weather, we also gathered daily weather information from the Japan Meteorological Agency website [31].

### Measurements

#### Restaurant sales data

Restaurant owners provided information on the prices of vegetable-rich meals and other meals offered, as well as the number of visitors and total sales per day.

#### SES

Our SES indicators were educational attainment, employment status, and average daily expenditures on lunchtime eating as a proxy for income or purchasing power. To measure educational attainment, we asked participants to state their highest level of education completed, categorised as follows: high school or lower, vocational/technical school or junior college, or 4-year college/university or higher. We classified participants’ employment status into four categories: regular employee, irregular employee, self-employed, and unemployed. To establish participants’ daily expenditures on lunchtime eating, we asked: “Which price range do you use for eating out or buying food for lunch on average? Please exclude occasions when you bring your lunch from home” and categorised responses into three groups: 450 yen or less, 451–850 yen, or 851 yen or more.

#### Covariates

Age, gender, day (weekday vs weekend), time surveyed, medical history, residence type, number of visits to the same restaurant during the same week, main objective of the visit, number of people in the respondent’s party, whether or not the participant was familiar with the Adachi “Eat vegetables daily” program, usual type of lunch, health consciousness about food, interest in eating vegetables, and daily weather data were compiled as potential covariates.

#### Sample size estimation

Based on preliminary interviews with restaurant staff, we assumed that the proportion of vegetable meal orders would be 15% during the control period and 25% during the intervention period. Based on this assumption, we calculated that a sample of 247 site-days per period would provide 80% power to detect a difference of at least 10 percentage points at a 5% significance threshold.

### Statistical analyses

After examining the restaurants’ characteristics and balance of customers with different characteristics between the control and intervention periods, we conducted three analyses. First, we calculated the covariate-adjusted difference and ratio regarding the proportion of vegetable-rich meal orders during the intervention period compared to the control period. Subsequently, to calculate the incidence ratio of vegetable-rich meal orders between the two data collection periods, we used a Poisson regression model with a log link function and robust standard error estimation for the data of a single business day as the unit of analysis [32]. When calculating daily sales, we subtracted the cost of cash-back payments from the restaurant’s total sales. We constructed a linear regression model using the log-transformed total sales with cash-back costs subtracted as the dependent variable and a dummy variable representing the intervention vs the control period as an explanatory variable. We estimated the ratio of and difference between the total sales and calculated the marginal means (i.e. the predicted values adjusted for the covariates mentioned above). Finally, we estimated the covariate-adjusted proportion of vegetable-rich meal orders according to individual characteristics and statistically evaluated between-SES differences in the difference between the two periods with the data at the individual level. We estimated denominator values, or the number of total daily customers, using the inverse of the sampling ratio from survey B as the frequency weight in our regression models (i.e. the number of non-vegetable-rich meal orders during the 1-h sampling period/the total number of non-vegetable-rich meal orders in each period reported by the restaurants; Additional file 1: Supplementary Material 1). Because our preliminary analysis showed that the effect-size gaps across SES were not clearly linear, we categorised SES into multiple groups rather than using continuous variable specifications. An intention-to-treat approach was used for all analyses. All analyses were performed using STATA version 14.2 (STATA Corp LP., College Station, Texas, USA). Further descriptions regarding the methods are available in Additional file 1: Supplementary Material 2.

## Results

The 26 participating restaurants (one dropped out) were diverse, including family and casual dining establishments, *Izakaya* (Japanese pubs), noodle shops, and those serving meals from various countries. The prices of vegetable-rich meals varied, ranging from 324 to 1450 yen (Table 1; Additional file 1: Supplementary Material 3). Among the 43 vegetable-rich meals served in participating restaurants, the price difference between the average vegetable-rich meal and the average regular meal was greater than 50 yen for 25 meals across 25 restaurants. The non-response rate for survey A (for all guests who ordered vegetable-rich meals) was 18.3% during the intervention period and 22.7% during the control period. The response rate for sampling survey B was 64% during the control period and 71% during the intervention period. In total, 511 respondents out of 7537 visitors (6.8%), and 704 respondents out of 7826 visitors (9.0%), ordered vegetable-rich meals during the control and intervention periods, respectively (Additional file 1: Supplementary Material 4 flow diagrams). The intra-class correlation coefficient was 0.112 (between-class variance: 0.007, standard error [SE]: 0.003; within-class variance: 0.056 and SE: 0.112). The average total sales per day were 73,157 yen during the control period (SE: 10,344) and 75,397 yen during the intervention period (SE: 11,078).

**Table 1:** Characteristics of participating restaurants

	Weekly business days	Type	Characteristics of vegetable-rich meals		
			Number of meals offered	Single dish or combo^a^	Price (Japanese yen)
A	5	Casual/family	1	Combo	950
B	6	Casual/family	1	Combo	1029
C	7	*Izakaya* pub	1	Single dish	680
D	6	Chinese	1	Combo	800
E	6	Italian	1	Combo	1000
F	4	Italian	4	Combo	880, 1080, 1280, 1450
G	5	Cafe	1	Combo	900
H	6	*Udon* noodle	1	Combo	830
I	7	*Izakaya* pub	3	Single dish, Combo	680 720, 780
J	6	Italian	1	Combo	880
K	5	Cafe	2	Combo	880
L	5	Casual/family	1	Combo	900
M	6	Western	2	Single dish	480, 560
N	7	Italian	1	Combo	930
O	6	Japanese	1	Single dish	600
P	6	*Izakaya* pub	1	Single dish	880
Q	7	*Ramen* noodle	1	Combo	850
R	6	*Ramen* noodle	2	Combo	950
S	6	Italian	5	Single dish	324, 432, 540, 756, 972
T	6	Chinese	3	Single dish	600, 1000, 1000
U	7	*Izakaya* pub	1	Single dish	680
V	6	Cafe	1	Combo	1300
W	6	Cafe	1	Combo	850
X	6	Casual/family	4	Combo	780, 780, 800, 880
Y	6	Chinese	2	Single dish, Combo	700 650
Z^b^	7	*Izakaya* pub	1	Single dish	650

The number of orders, visitors and total sales per day in each restaurant are shown in Additional file 1: Supplementary Material 3

^a^Single dish salad-type meal or combo-type meal

^b^Dropped out

Compared to the control period, the crude proportion of vegetable-rich meal orders per day during the intervention period was 1.33 times higher (95% confidence interval [CI]: 1.18 to 1.49; Table 2), and when covariates were adjusted, the value increased to 1.50 (95% CI: 1.29 to 1.75). The crude total sales were also 1.14 times higher (95% CI: 0.63 to 2.05), and when all covariates were adjusted, the value increased to 1.77 (95% CI: 1.11 to 2.83), which was equivalent to an average daily revenue gain of 14,423 yen, even when subtracting the 50-yen cash back (Table 2).

**Table 2:** Ratios of vegetable-rich meal orders and daily restaurant sales (during the intervention period vs the control period)

	Non-adjusted	Model 1	Model 2
Vegetable-rich meal orders			
Intervention (ref. control)	1.33 (1.18, 1.49)	1.35 (1.21, 1.52)	1.50 (1.29, 1.75)
Weekends (ref. weekdays)			1.18 (0.98, 1.42)
Temperature (per 1 °C increase)			0.91 (0.86, 0.97)
Humidity (per 10% point increase)			1.06 (0.99, 1.13)
Weather: Rain (ref. not rain)^a^			0.89 (0.74, 1.08)
Adjusted for fixed effects of restaurants	No	Yes	Yes
Restaurant sales			
Intervention (ref. control)	1.14 (0.63, 2.05)	1.15 (0.82, 1.61)	1.77 (1.11, 2.83)
Weekends (ref. weekdays)			1.22 (0.75, 1.98)
Temperature (per 1 °C increase)			0.81 (0.69, 0.96)
Humidity (per 10% point increase)			0.92 (0.78, 1.07)
Weather: Rain (ref. no rain)^a^			0.70 (0.44, 1.12)
Adjusted for fixed effects of restaurants	No	Yes	Yes

^a^Based on weather during business hours. We estimated the ratios for vegetable-rich meal orders and restaurant sales separately. Model 1 adjusted for fixed effects of restaurants by adding the dummy variables identifying restaurants. In Model 2, we further added daily temporal and climatic data as covariates

Vegetable-rich meals were ordered by 356 respondents out of 6301 visitors (5.7%) during the control period and 456 out of 6650 visitors (6.9%) during the intervention period (Table 3). Compared to the control period, during the intervention period, the visitors at the participating restaurants were younger, were female, were likely to have a history of serious medical conditions, were non-Adachi Ward residents, were university/college graduates, were regular employees, had lower average lunch expenditures, were likely to visit with others (not mainly for a meal), and were less interested in eating vegetables (Table 3).

**Table 3:** Proportion of vegetable-rich meal orders, *n* (%)^a^ by individual characteristics during the intervention and control periods

	Control period		Intervention period	
	Total (*n* = 6301)	Vegetable-rich meal orders (*n* = 356)	Total (*n* = 6650)	Vegetable-rich meal orders (*n* = 456)
Age (years)				
34 or below	908 (14.4)	88 (9.7)	1654 (24.9)	79 (4.8)
35–49	892 (14.2)	117 (13.1)	1270 (19.1)	128 (10.1)
50–64	2192 (34.8)	67 (3.1)	2702 (40.6)	118 (4.4)
65 or above	2267 (36.0)	70 (3.1)	1012 (15.2)	119 (11.8)
Female	2478 (39.3)	206 (8.3)	4046 (60.8)	315 (7.8)
Has a history of serious medical conditions	2332 (37.0)	108 (4.6)	3250 (48.9)	147 (4.5)
Resident in the Adachi Ward	3626 (57.5)	208 (5.7)	2862 (43.0)	288 (10.1)
Educational attainment				
High school or less	2141 (34.0)	116 (5.4)	1673 (25.2)	170 (10.2)
Vocational/technical school/junior college	603 (9.6)	81 (13.4)	585 (8.8)	97 (16.6)
University/college or higher	2826 (44.9)	133 (4.7)	4151 (62.4)	150 (3.6)
Employment status				
Regular employee	2530 (40.2)	168 (6.6)	3561 (53.5)	166 (4.7)
Irregular employee	1512 (24.0)	53 (3.5)	778 (11.7)	97 (12.5)
Self-employed	77 (1.2)	33 (42.9)	237 (3.6)	32 (13.5)
Unemployed	1514 (24.0)	86 (5.7)	2010 (30.2)	132 (6.6)
Average lunch expenditures per day				
450 yen or less	1580 (25.1)	29 (1.8)	2009 (30.2)	50 (2.5)
451–850 yen	2477 (39.3)	137 (5.5)	2901 (43.6)	175 (6.0)
851 yen or more	2233 (35.4)	179 (8.0)	1617 (24.3)	210 (13.0)
Participating in the campaign twice or more during one period	53 (0.8)	18 (34.0)	478 (7.2)	36 (7.5)
Visiting with other(s)	3083 (48.9)	235 (7.6)	4248 (63.9)	341 (8.0)
Visiting not mainly for a meal	2980 (47.3)	21 (0.7)	1190 (17.9)	42 (3.5)
Familiar with the “Adachi *beji-tabe-raifu*” program before the visit				
Yes	4880 (77.4)	263 (5.4)	5816 (87.5)	276 (4.7)
No	842 (13.4)	82 (9.7)	786 (11.8)	162 (20.6)
Usual type of lunch				
Homemade (boxed lunch)	2549 (40.5)	133 (5.2)	1776 (26.7)	216 (12.2)
Bought at retail shops	543 (8.6)	76 (14.0)	1370 (20.6)	72 (5.3)
Eaten at a restaurant	3072 (48.8)	114 (3.7)	3264 (49.1)	131 (4.0)
Consider health when selecting food/meals				
Always, often	5052 (80.2)	300 (5.9)	4898 (73.7)	386 (7.9)
Not often, rarely	1245 (19.8)	52 (4.2)	1696 (25.5)	59 (3.5)
Interest in eating vegetables				
High	2312 (36.7)	140 (6.1)	2441 (36.7)	224 (9.2)
Low	3352 (53.2)	185 (5.5)	4095 (61.6)	183 (4.5)

^a^The percentage of “Total” column is the column percentage, and the “Vegetable-rich meal orders” column is the row percentage, respectively

**Fig. 1:**
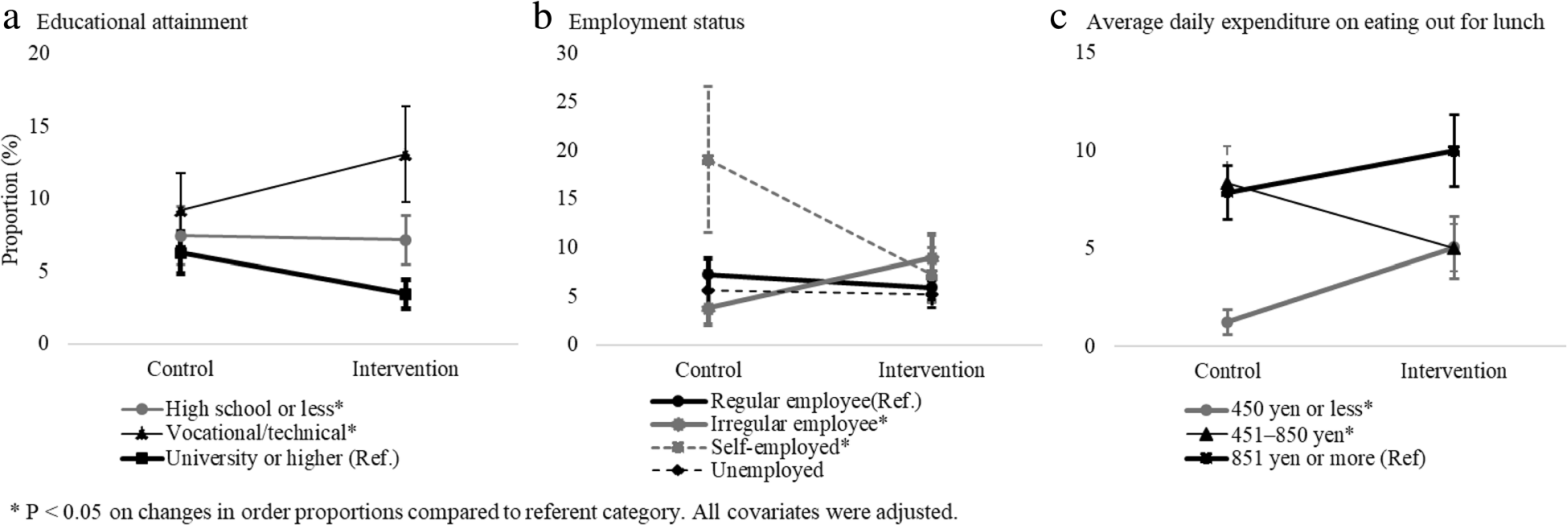
Covariate-adjusted proportion of vegetable-rich meal orders. **a** Educational attainment. **b** Employment status. **c** Average daily expenditure on eating out for lunch

When adjusting for all covariates, including restaurant dummy codes, compared with the control period, vegetable-rich meal orders among higher educated patrons decreased by 2.8 percentage points (95% CI: − 3.31 to − 2.37) during the intervention period, while the decrease was only 0.3 percentage points (95% CI: − 0.59 to 0.04) among those with a high school education or less (*P* for interaction = 0.041). Among vocational/technical school graduates, the proportion increased by 3.9 percentage points (95% CI: 3.06 to 4.63, *P* for interaction < 0.001; Fig. 1a). For these vocational/technical school graduates, individuals who tended to show an increase in their proportion of vegetable-rich meal orders were more likely to be over the age of 65, female, irregular employees or unemployed, visiting the restaurant not mainly for the purpose of a meal, and reported cooking lunch regularly (data not shown).

The change in vegetable-rich meal orders (percentage points) was − 1.4 among regular employees (95% CI: − 1.66 to − 1.05), + 5.2 among irregular employees (95% CI: 4.54 to 5.76, *P* for interaction = 0.001), and − 11.9 among self-employed patrons (95% CI: − 16.54 to − 7.19, *P* for interaction = 0.013; Fig. 1b). The change in vegetable-rich meal orders among customers who usually paid the most (850+ yen) to eat out for lunch was 2.1 percentage points (95% CI: 1.66 to 2.62), while those who reported that they paid the least (450 yen or less) increased their vegetable-rich orders by 3.8 percentage points (95% CI: 2.82 to 4.76, *P* for interaction = 0.001). The change among those who reported spending an intermediate amount (451–850 yen) was − 3.3 (95% CI: − 3.99 to − 2.65, *P* for interaction = 0.001; Fig. 1c).

## Discussion and conclusions

As hypothesised, the intervention program increased the number of visitors to restaurants by 1.04 times, and among the visitors, the total proportion of vegetable-rich meal orders also increased by 1.5 times. This resulted in increased daily restaurant sales during the intervention period. The overall success of this campaign (i.e. an increase in the number of visitors to the participating restaurants and people choosing healthier meals) is consistent with recent intervention trials using positive incentives for health behaviour [18]. The intervention was most effective among the group with the lowest purchasing power, who exhibited the largest increase in their likelihood of ordering vegetable-rich meals.

The equalising impact of the intervention was most notable when analysing effect sizes based on participants’ financial conditions. Our results were not consistent with those from a supermarket-based randomised controlled trial that provided financial incentives for recommended food in New Zealand. This trial revealed no difference in the effect based on customers’ income and education [24]. A potential explanation for this inconsistency could be differences in the settings of each intervention (supermarkets vs restaurants). Customers at restaurants, as in the present study, usually select and consume meals for themselves, while foods purchased at supermarkets are not necessarily consumed by the purchaser. Moreover, there also exists a longer time lag between the action (taking food from the supermarket shelves) and consumption in a retail setting compared to a restaurant, in which customers eat the meal immediately after being served. Marketing research suggests that immediate and direct rewards are more effective in securing customers’ loyalty (i.e. repeated purchases) [33, 34].

Financial incentives could encourage people to engage in the targeted behaviour by two mechanisms. First, this type of incentive encourages people to change their considered decisions by making up the difference between the utility values for each choice. If a regular meal costs 600 yen, and a particular individual values an added vegetable serving at 100 yen, then a 100-yen cash-back campaign is sufficient for encouraging the individual to purchase the meal. Alternatively, an individual may purchase the vegetable serving, even if the discount is less than 100 yen—say, 50 yen, as in our intervention—if the time-limited cash-back offer stimulates the individual’s affect (i.e. a sense of a perceived “deal”, loss-aversion, or enjoyment). The latter is in line with the concept of nudging [13]. In our study, 25 out of 43 vegetable-rich meal options were more expensive than regular meals by more than 50 yen. In such cases, those who ordered the vegetable-rich meals paid extra money for those vegetables.

We should note the limitations of this study. First and foremost, although we had strong advantages with our study design as mentioned above, the non-parallel design is prone to selection bias. For example, the results of our adjusted estimates for total vegetable-rich orders and restaurant sales were, when considering the weather, day of the week, and restaurant, fixed effects that were larger than the crude estimates. This may be due to the strong confounding factors, as the intervention was implemented in the unstable rainy season. However, residual confounding is also possible. In fact, compared to the control period, during the intervention period, visitors to the participating restaurants were more likely to be young, female, university graduates, and regular employees. They were basically less likely to order vegetable-rich meals in the intervention period, potentially resulting in an underestimation of the impacts of the intervention on vegetable-rich meal orders. Meanwhile, the intervention also involved more non-Adachi residents, unemployed and poorer individuals (paying less for lunch), people visiting with someone, and people not visiting mainly for a meal. They were more likely to order vegetable-rich meals in the campaign, potentially resulting in an overestimation of the intervention effects. To confirm the findings of this study, future studies should implement a cluster randomised trial, allocating multiple restaurants to two groups simultaneously. Because a pure randomised trial would also have critical limitations (e.g. on generalisability, feasibility), both our real-world effectiveness study and future randomised efficacy study would contribute to causal inferences [35, 36]. Second, we could not control all the behaviours of the restaurant staff members, which introduces bias to our intervention. For example, restaurant staff members may have provided cash back to those who did not order vegetable-rich meals. However, we employed an intention-to-treat analysis, which evaluates the effectiveness of an intervention in a conservative way. Non-involvement among eligible customers was around 20% in both the intervention and control periods, suggesting that any bias due to non-participation was trivial. Third, repeat visitors to a restaurant, who knew the cash-back incentive was available the next week, might have held back on ordering vegetable-rich meals during the control period. However, the gap in percentage points for repeated orders was only 2.8 (5.1% during the control period and 7.9% during the intervention period). Fourth, the actual effect size (the vegetable order gap was 2.2%) was much smaller than the gap we assumed based on our sample size calculation (10%), indicating that our study was underpowered. This may explain some counter-intuitive results, including the reduction in the orders of vegetable-rich meals among some subgroups in the intervention period. Given the actual effect size observed, the data required 2317 site-days based on the same statistical power and rejection rate. A larger-sized intervention is warranted in the future. Finally, despite the wide range of meal types among our participating restaurants, our representativeness of the full range of restaurants and types of eating-out venues is questionable. For example, our participating restaurants did not include popular fast food chains.

In conclusion, even a low-value monetary incentive offered over a limited period was able to increase the proportion of healthy meal orders across local restaurants. This type of intervention could encourage subgroups with low incomes and irregular employments to make healthy dietary choices. Although our study suggested potentially stronger impacts for more financially disadvantaged people, the proportional impact for other dimensions of SES was not very clear. Further analyses may help to better evaluate the impact on population subgroups with not just a single factor (e.g. income) but through multiple factors, using cluster analysis to identify subgroups with specific combinations of characteristics (e.g. low-income, male college students).

Since a single incentive might not be effective in the long run, future studies should aim to identify and evaluate similar low-cost behaviour-change options that could contribute to the design of more comprehensive and continuous interventions. Moreover, behavioural economics studies have suggested that chronic social stresses due to the scarcity of money or time could restrict cognitive function and discount the value of the benefits obtained in the future [37, 38]. Given that, it is possible that socioeconomically disadvantaged people are less likely to engage in a health-maintaining behaviour (e.g. eating more vegetables) because that is an investment in a better future life. Thus, if financial incentive programmes for healthy choices are not enough to attract people enduring social stresses, such participants could drop-out of programmes early, or not participate in the programmes at all, which, in turn, could lead to a widening of long-term health inequalities. Another concern about our intervention is that financially disadvantaged people who cannot afford to eat out do not benefit from the intervention. Hence, when developing incentive intervention programmes for health behaviours, it is essential to undertake careful long-term evaluations of the programmes according to social subgroups and create necessary modifications to make the intervention programmes effective, feasible, and sustainable in reducing inequality in the targeted health behaviours [14]. Creating a knowledge base of various behavioural approaches should contribute to global strategies geared toward addressing health inequalities.

## Additional file

Additional file 1: **Supplementary Material 1.** Frequency weights calculation. **Supplementary Material 2.** Additional descriptions on study design and analysis. **Supplementary Material 3.** The number of orders, visitors and the total takings per day in each restaurant. **Supplementary Material 4.** Flow diagrams of participating individuals and restaurants. (DOCX 88 kb)

## Data Availability

The datasets generated and/or analysed during the current study are not publicly available due utilisation of individual data from this study is generally not permitted by the Government of Adachi Ward, but are available from the corresponding author on reasonable request.
