# Supplementary material for: Effectiveness of a low-value financial-incentive program for increasing vegetable-rich restaurant meal selection and reducing socioeconomic inequality: a cluster crossover trial
Source: Int J Behav Nutr Phys Act. 2019 Sep 12;16:81. doi: 10.1186/s12966-019-0830-5 (PMC6740003; doi:10.1186/s12966-019-0830-5)
Supplement: Supplementary file 1 — Supplementary Material 1. Frequency weights calculation. Supplementary Material 2. Additional descriptions on study design and analysis. Supplementary Material 3. The number of orders, visitors and the total takings per day in each restaurant. Supplementary Material 4. Flow diagrams of participating individuals and restaurants. (DOCX 88 kb) [file 12966_2019_830_MOESM1_ESM.docx]

**Additional file 1**

**The effectiveness of a low-value financial incentives program for reducing socioeconomic inequality by selecting vegetable-rich restaurant meals: A cluster crossover trial**

**International Journal of Behavioral Nutrition and Physical Activity**

**Wataru NAGATOMO**

Department of Health Education and Health Sociology, The University of Tokyo, 7-3-1 Hongo, Bunkyo-ku, Tokyo 113-0033, Japan

Postgraduate Student

wnagatomo-tky@umin.ac.jp

**Junko SAITO**

Department of Health Education and Health Sociology, The University of Tokyo, 7-3-1 Hongo, Bunkyo-ku, Tokyo 113-0033, Japan

Postdoctoral Fellow

j.junkosaito@gmail.com

**Naoki KONDO***

Department of Health Education and Health Sociology, School of Public Health, The University of Tokyo, 7-3-1 Hongo, Bunkyo-ku, Tokyo 113-0033, Japan

Associate Professor

naoki-kondo@umin.ac.jp

***Corresponding author**

# **Supplementary Material 1.** Frequency weights calculation

| Restaurants | Total customers (A) | | #N vegetable-rich meal orders (B) | | #N not ordering vegetable-rich meal (C = A - B) | | #N not ordering vegetable-rich meal in Survey B: 1-hour random sample (D) | | Frequency weights (D/C) | |
| --- | --- | --- | --- | --- | --- | --- | --- | --- | --- | --- |
|  |  |  |  |  |  |  |  |  |  |  |
|  |  |  |  |  |  |  |  |  |  |  |
|  | Control | Intervention | Control | Intervention | Control | Intervention | Control | Intervention | Control | Intervention |
| A | 131 | 180 | 20 | 43 | 111 | 137 | 4 | 9 | 28 | 15 |
| B | 77 | 68 | 12 | 25 | 65 | 43 | 2 | 5 | 33 | 9 |
| C | 633 | 773 | 11 | 14 | 622 | 759 | 0 | 3 | ‐ | 253 |
| D | 98 | 116 | 1 | 6 | 97 | 110 | 4 | 8 | 24 | 14 |
| F | 108 | 98 | 6 | 34 | 102 | 64 | 4 | 3 | 26 | 21 |
| H | 429 | 373 | 47 | 61 | 382 | 312 | 11 | 9 | 35 | 35 |
| J | 159 | 179 | 5 | 7 | 154 | 172 | 1 | 2 | 154 | 86 |
| L | 170 | 243 | 22 | 18 | 148 | 225 | 1 | 5 | 148 | 45 |
| M | 108 | 94 | 6 | 10 | 102 | 84 | 3 | 0 | 34 | ‐ |
| N | 2785 | 2682 | 120 | 109 | 2665 | 2573 | 5 | 3 | 533 | 858 |
| P | 140 | 148 | 5 | 3 | 135 | 145 | 0 | 5 | ‐ | 29 |
| Q | 629 | 621 | 36 | 35 | 593 | 586 | 2 | 0 | 297 | ‐ |
| R | 560 | 526 | 28 | 17 | 532 | 509 | 10 | 10 | 53 | 51 |
| V | 39 | 41 | 12 | 10 | 27 | 31 | 4 | 2 | 7 | 16 |
| W | 12 | 27 | 0 | 14 | 12 | 13 | 4 | 0 | 3 | ‐ |
| X | 991 | 1193 | 41 | 88 | 950 | 1105 | 13 | 9 | 73 | 123 |

Frequency weights were rounded off to the nearest integer.

# **Supplementary Material 2.** Additional descriptions on study design and analysis

The government runs a program entitled “Eat vegetables daily”(in Japanese: *Adachi beji-tabe-raifu*). Given the effectiveness of a higher intake of vegetables[1–3] and of eating vegetables before carbohydrates[4] in preventing diabetes and cardiovascular disease, the program was put in place as a part of a set of diabetes countermeasures, and aims to engineer physical and social environments such that people naturally or unconsciously increase their intake of vegetables, particularly targeting socioeconomically vulnerable populations[1,2]. vegetable-rich meals contained at least 120g or more of vegetables per serving. Restaurants can also be certified by serving vegetable-first meals. These are meals accompanied by a salad, or other vegetable dish, that must be served before the main dish. Such vegetable-rich and vegetable-first meals (hereinafter referred to collectively as “vegetable-rich meals”) were regularly available independent of this specific study. We excluded restaurants where vegetable-rich meals were provided to all customers. Participants in the study were customers of these restaurants during the survey period who completed our questionnaires.

To collect information on individuals who ordered vegetable-rich meals, the first survey targeted all customers who ordered such meals during both the control and the intervention periods (Survey A, administered to all customers ordering vegetable-rich meals). To calculate the proportion of vegetable-rich meals ordered according to individuals’ backgrounds, we needed denominator data: i.e., total number of customers by socioeconomic status. To acquire these data, we conducted another survey targeting all customers, including those who did not order vegetable-rich meals. To minimize survey cost, this was carried out only for specific samples. We surveyed the customers who visited during a randomly-selected one-hour period during both the control and intervention periods in each restaurant (Survey B). While the restaurant staff conducted the surveys of customers who ordered vegetable-rich meals, one or two researchers visited each restaurant and directly asked the other customers (those who did not order vegetable-rich meals) to answer a questionnaire.

For use as covariates, we measured age (4 categories), gender (2 categories), day (weekday vs. weekend) and time surveyed (lunchtime (noon–2PM) vs. other), medical history, residency in Adachi Ward, the number of visits to the same restaurant during the same week (once vs. twice or more), main objective of visit (eating vs. other), number of people in the respondent’s party (dichotomized as visiting with other(s) vs. alone), whether or not the participant was familiar with the Adachi “Eat vegetables daily" program, usual type of lunch (homemade vs retail shop bought vs. restaurant meal), health consciousness about food, and interest in eating vegetables. To measure interest in eating vegetables, we asked four questions: "When you eat out, how often do you order vegetable-rich meals?", answered by selecting from 4 Likert-type response options from 0 for "none" to 1 for "always"; "How many times per day do you eat vegetable dishes?", answered by selecting from 3 response options scored from 0 for "once or none" to 1 for “three times"; and "Did you know that the required vegetable intake for adults is over 350 grams per day?" and "Do you eat vegetables first regularly?", each answered by selecting from 2 response options scored 0 for "no" or 1 for "yes". The participant’s scores on each question were summed to calculate the total, and dichotomized as high vs. low using a median split. Daily weather information gathered included average temperature (degrees Celsius), average humidity (%), and whether the weather was categorised as “not rainy” or “rainy”.

To test the first two hypotheses of this study, we calculated the difference between and ratio of the total number of vegetable-rich meal orders and total daily restaurant takings in the intervention period compared to the control period. To test the third hypothesis, we calculated the proportion of vegetable-rich meal orders per restaurant per day and compared the difference in this proportion between the intervention and control periods according to customers’ SES.

After examining the restaurants’ characteristics and the balance of customers with different characteristics between the control and intervention periods, we conducted three analyses. First, we calculated the covariate-adjusted difference between and ratio of the proportion of vegetable-rich meals orders during the intervention period compared to the control period. Subsequently, to calculate the incidence ratio of vegetable-rich meal orders between the two data collection periods, we used a Poisson regression model with log link function and robust standard error estimation, following recent statistical recommendations for modelling a frequent event (occurring in more than 10% of cases)[5]. We adjusted for dummy variables representing the restaurant, weekday vs. weekend, temperature, humidity, and rain. We used the log-transformed number of customers per day as an offset value, and estimated the restaurant’s total takings per day per restaurant in the same way. When calculating daily takings, we subtracted the cost of paying cashback from the restaurant’s total takings so that the results would be applicable to a regular, non-research campaign by restaurant owners who may pay these costs themselves. We constructed a linear regression model using the log-transformed total takings with cashback costs subtracted as the dependent variable and a dummy variable representing intervention vs. control period as an explanatory variable. We estimated the ratio of and difference between total takings and calculated the marginal means, i.e., the predicted values adjusting for the covariates mentioned above.

Finally, we estimated the covariate-adjusted proportion of vegetable-rich meal orders according to individual characteristics, and statistically evaluated between-SES differences in the difference between the two periods. For numerator values, we used the data of all customers who ordered vegetable-rich meals. Among covariates, we performed difference-in-difference analysis using weighted Poisson regression with SES and a dummy variable representing intervention vs. control period as explanatory variables. Because our preliminary analysis showed that the effects according to participants’ levels of educational attainment were not intuitively interpretable, for interpretation purposes, we further tested for differences in the proportion of vegetable-rich meal orders by participants’ educational characteristics. An intention-to-treat approach was used for all analyses. All analyses were performed using STATA version 14.2 (STATA Corp LP., College Station, Texas, USA).

References

[1] Bazzano LA. Dietary intake of fruit and vegetables and risk of diabetes mellitus and cardiovascular diseases. World Health Organization; 2005.

[2] Lim SS, Vos T, Flaxman AD, Danaei G, Shibuya K, Adair-Rohani H, et al. A comparative risk assessment of burden of disease and injury attributable to 67 risk factors and risk factor clusters in 21 regions, 1990-2010: a systematic analysis for the Global Burden of Disease Study 2010. Lancet 2012;380:2224–60. doi:10.1016/S0140-6736(12)61766-8.

[3] Nagura J, Iso H, Watanabe Y, Maruyama K, Date C, Toyoshima H, et al. Fruit, vegetable and bean intake and mortality from cardiovascular disease among Japanese men and women: the JACC Study. Br J Nutr 2009;102:285–92. doi:10.1017/S0007114508143586.

[4] Imai S, Fukui M, Kajiyama S. Effect of eating vegetables before carbohydrates on glucose excursions in patients with type 2 diabetes. J Clin Biochem Nutr 2014;54:7–11. doi:10.3164/jcbn.13-67.

[5] Barros AJD, Hirakata VN. Alternatives for logistic regression in cross-sectional studies: an empirical comparison of models that directly estimate the prevalence ratio. BMC Med Res Methodol 2003;3:21. doi:10.1186/1471-2288-3-21.

# **Supplementary Material 3.** The number of orders, visitors and the total takings per day in each restaurant

| Restaurants | Operation data per day^1^：Mean [SD] | | | | | |
| --- | --- | --- | --- | --- | --- | --- |
|  | The number of people ordering  vegetable-rich meals | | The number of visitors | | Total takings (yen) | |
|  | Control | Intervention | Control | Intervention | Control | Intervention |
| A | 3.3 [3.1] | 7.2 [3.3] | 26.2 [6.2] | 36.0 [8.3] | ‐ | ‐ |
| B | 2.0 [2.4] | 4.2 [4.2] | 12.8 [13.1] | 11.3 [8.2] | ‐ | ‐ |
| C | 1.4 [1.3] | 1.8 [1.6] | 90.4 [35.7] | 110.4 [51.9] | 277 534 [120 608] | 305 221 [145 268] |
| D | 0.2 [0.4] | 1.0 [1.3] | 16.3 [6.6] | 19.3 [8.1] | 20 955 [2069] | 26 872 [11 719] |
| E | 17.1 [7.3] | 15.6 [6.4] | 25.5 [9.1] | 21.5 [8.0] | 26 847 [10 025] | 23 167 [8991] |
| F | 1.2 [0.4] | 6.8 [4.8] | 27.0 [8.2] | 24.5 [13.2] | 38 575 [16 762] | 32 073 [15 443] |
| G | 7.2 [2.4] | 14.6 [2.3] | 39.6 [14.3] | 54.0 [5.7] | 41 734 [13 173] | 61 298 [5581] |
| H | 6.7 [4.5] | 8.7 [5.0] | 71.5 [15.6] | 62.2 [7.9] | 70 943 [19 328] | 63 873 [5850] |
| I | 0.7 [0.8] | 1.6 [2.1] | ‐ | ‐ | ‐ | ‐ |
| J | 0.8 [1.3] | 1.2 [1.0] | 16.2 [4.9] | 18.0 [6.1] | 16 023 [4712] | 21 648 [10 253] |
| K | 4.0 [3.0] | 2.7 [2.7] | ‐ | ‐ | - | - |
| L | 7.3 [6.5] | 6.0 [3.6] | 48.0 [37.7] | 53.3 [37.3] | 41 413 [30 857] | 50 143 [37 076] |
| M | 1.2 [0.8] | 2.0 [1.6] | 23.0 [8.1] | 16.8 [7.5] | 29 682 [7128] | 37 938 [23 088] |
| N | 15.0 [4.0] | 13.6 [7.3] | 397.9 [37.2] | 383.1 [65.3] | 167 348 [31 293] | 162 993 [39 842] |
| O | 0.2 [0.4] | 1.3 [0.5] | ‐ | ‐ | ‐ | ‐ |
| P | 0.8 [1.3] | 0.5 [0.5] | 23.3 [12.9] | 24.7 [13.7] | ‐ | ‐ |
| Q | 4.5 [1.5] | 4.4 [3.5] | 89.9 [23.8] | 88.7 [19.7] | ‐ | ‐ |
| R | 4.7 [2.0] | 2.8 [1.7] | 93.3 [12.5] | 87.7 [10.1] | ‐ | ‐ |
| S | 1.0 [1.0] | 1.7 [1.7] | 14.0 [9.1] | 13.7 [8.2] | 38 520 [26 339] | 40 010 [15 335] |
| T | 0 | 0.3 [0.5] | ‐ | ‐ | ‐ | ‐ |
| U | 0.1 [0.4] | 0.7 [1.5] | 7.4 [1.4] | 4.3 [1.6] | 16 510 [3443] | 9973 [4779] |
| V | 2.4 [2.3] | 2.0 [1.2] | 7.8 [4.2] | 6.6 [2.4] | 10 852 [4706] | 8082 [2865] |
| W | 0 | 2.3 [2.9] | 2.0 [3.2] | 4.5 [6.0] | 2103 [2744] | 6248 [7246] |
| X | 5.9 [3.9] | 12.6 [6.3] | 165.2 [32.8] | 198.8 [38.7] | 278 414 [41 090] | 284 564 [102 690] |
| Y | 0.8 [0.8] | 1.8 [0.8] | 7.2 [7.1] | 4.0 [2.0] | 10 933 [14 153] | 3608 [2048] |
| Z³ | 0 | 0 | ‐ | ‐ | ‐ | ‐ |
| ^1^ The number of questionnaire respondents was used for orders. Information on the number of visitors was requested to each restaurant, but it was missing for some restaurants that did not receive a response. | | | | | | |

# **Supplementary Material 4.** Flow diagrams of participating individuals and restaurants

**For the analysis comparing the proportion of vegetable meal orders (unit: day)**

156 days in each period in 26 restaurants

7 days lost, from1 restaurant dropped out

24 days lost, from 4 restaurant not providing the number of total visitors

125 days in each period in 21 restaurants

3 days in control and 1 day in intervention excluded: temporary closure

1 day in each and 2 days in intervention: no visitor

1 day in intervention: missing

119 days in control period: 7537 visitors including 511 vegetable meal orders

122 days in intervention period: 7826 visitors including 704 vegetable meal orders

**For the analysis comparing the proportion of total takings per day (unit: day)**

156 days in each period in 26 restaurants

54 days excluded as 10 restaurants not providing the total takings information

95 days in each period in 16 restaurants

3 days in control and 1 day in intervention periods excluded due to temporary closure

92 days in control period

94 days in intervention period

**Survey B.** Participants visiting restaurants in the randomly selected one hour.

26 restaurants

5 participants excluded as they visited the restaurants when no non-vegetable meal orders (4 restaurants); 16 participants in a restaurant excluded as the restaurant did not provide the number of visitors per day

16 restaurants

78 participants in control period

107 participants in intervention period

20 restaurants

83 participants in control period

123 participants in intervention period

1 dropped out; 5 refused Survey B

25 restaurants

Control period: 571 participants

Intervention period: 765 participants

1 dropped out

26 restaurants

**Survey A.** Participants ordering vegetable meal.

16 restaurants

Control period: 356 participants

Intervention period: 456 participants

Excluded due to not participating in Survey B

**For the analysis comparing the proportion of vegetable meal orders by individual SES**
